# Supplementary material for: Betacyanins from red pitahaya (Hylocereus polyrhizus) exhibit antiviral response against influenza A virus
Source: Heliyon. 2024 Jun 15;10(12):e33049. doi: 10.1016/j.heliyon.2024.e33049 (PMC11252771; doi:10.1016/j.heliyon.2024.e33049)
Supplement: Multimedia component 1 [file mmc1.docx]

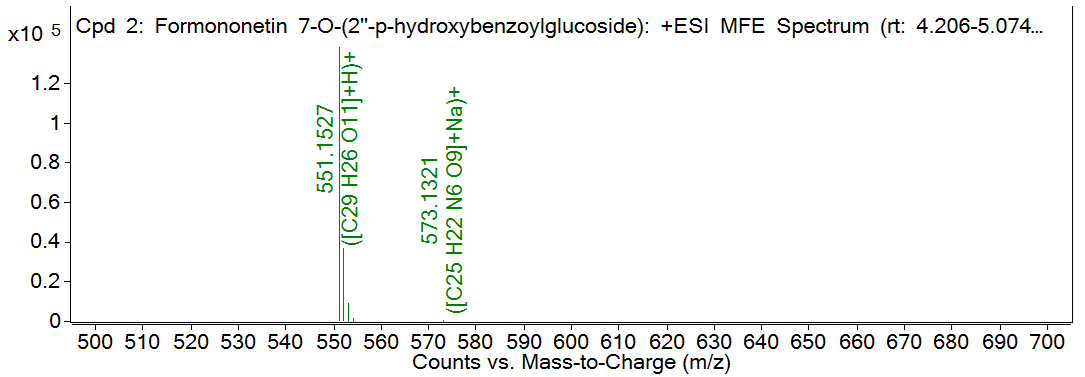


**Fig S1.** ESI-Q-TOF-MS spectrum of betanin (*m/z* 551 [M + H]^+^) at retention time 4.483 min.


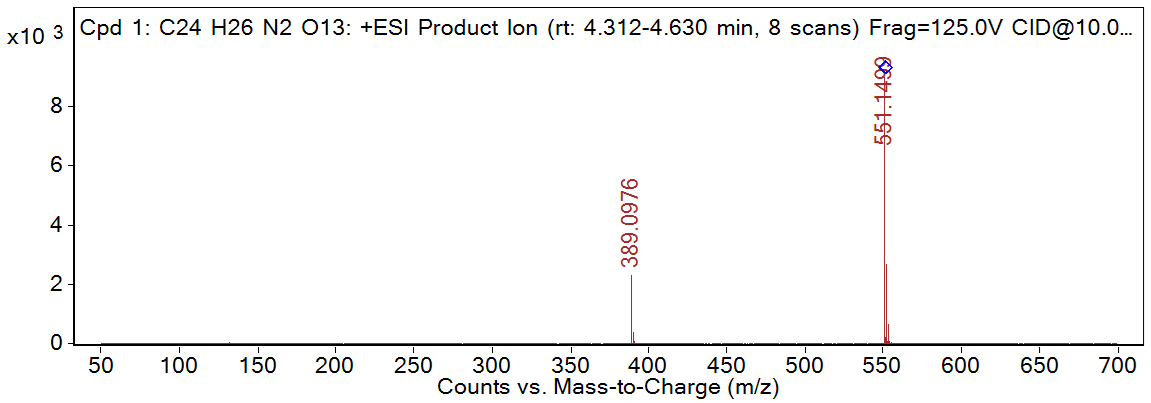


**Fig S2.** ESI-Q-TOF-MS/MS spectrum of betanin (*m/z* 551 [M+H]^+^) at retention time 4.483 min.


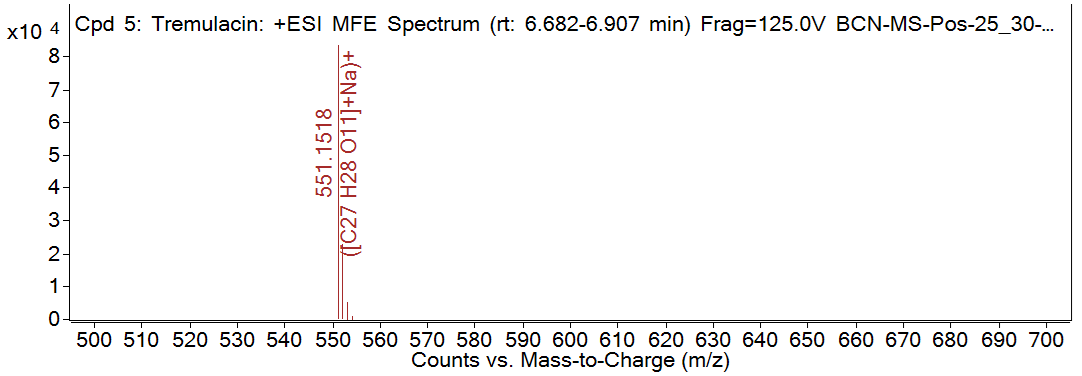


**Fig S3.** ESI-Q-TOF-MS spectrum of isobetanin (*m/z* 551 [M+H]^+^) at retention time 6.749 min.


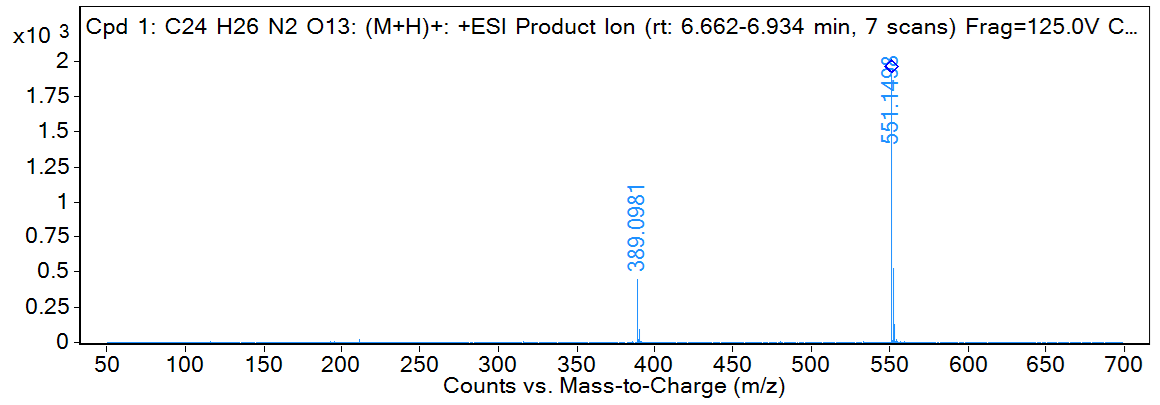


**Fig S4.** ESI-Q-TOF-MS/MS spectrum of isobetanin (*m/z* 551 [M+H]^+^) at retention time 6.749 min.


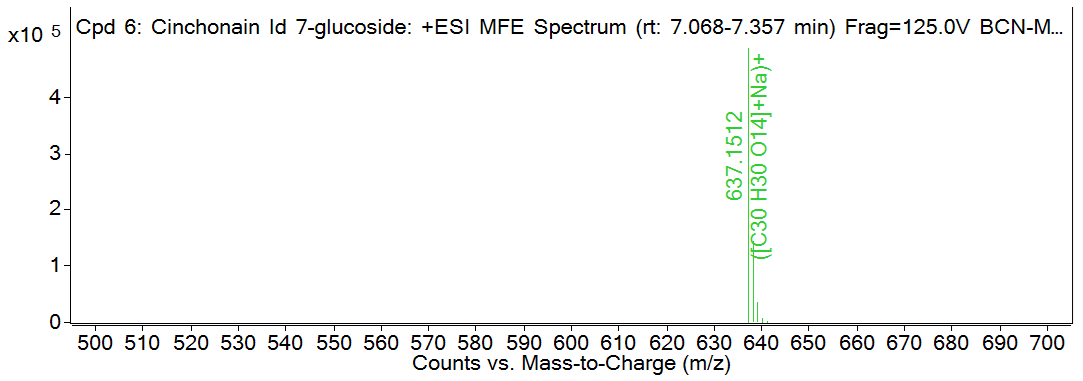


**Fig S5.** ESI-Q-TOF-MS spectrum of phyllocactin (*m/z* 637 [M+H]^+^) at retention time 7.189 min.


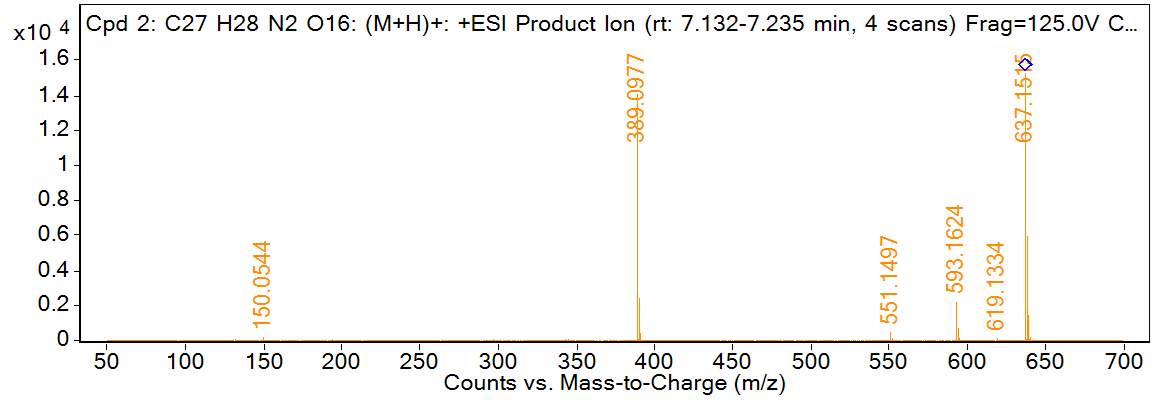


**Fig S6.** ESI-Q-TOF-MS/MS spectrum of phyllocactin (*m/z* 637 [M+H]^+^) at retention time 7.189 min.


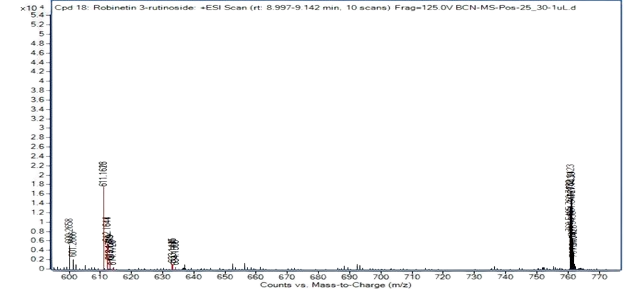


**Fig S7.** ESI-Q-TOF-MS spectrum of hylocerenin (*m/z* 695 [M+H]^+^) at retention time 9.044 min.


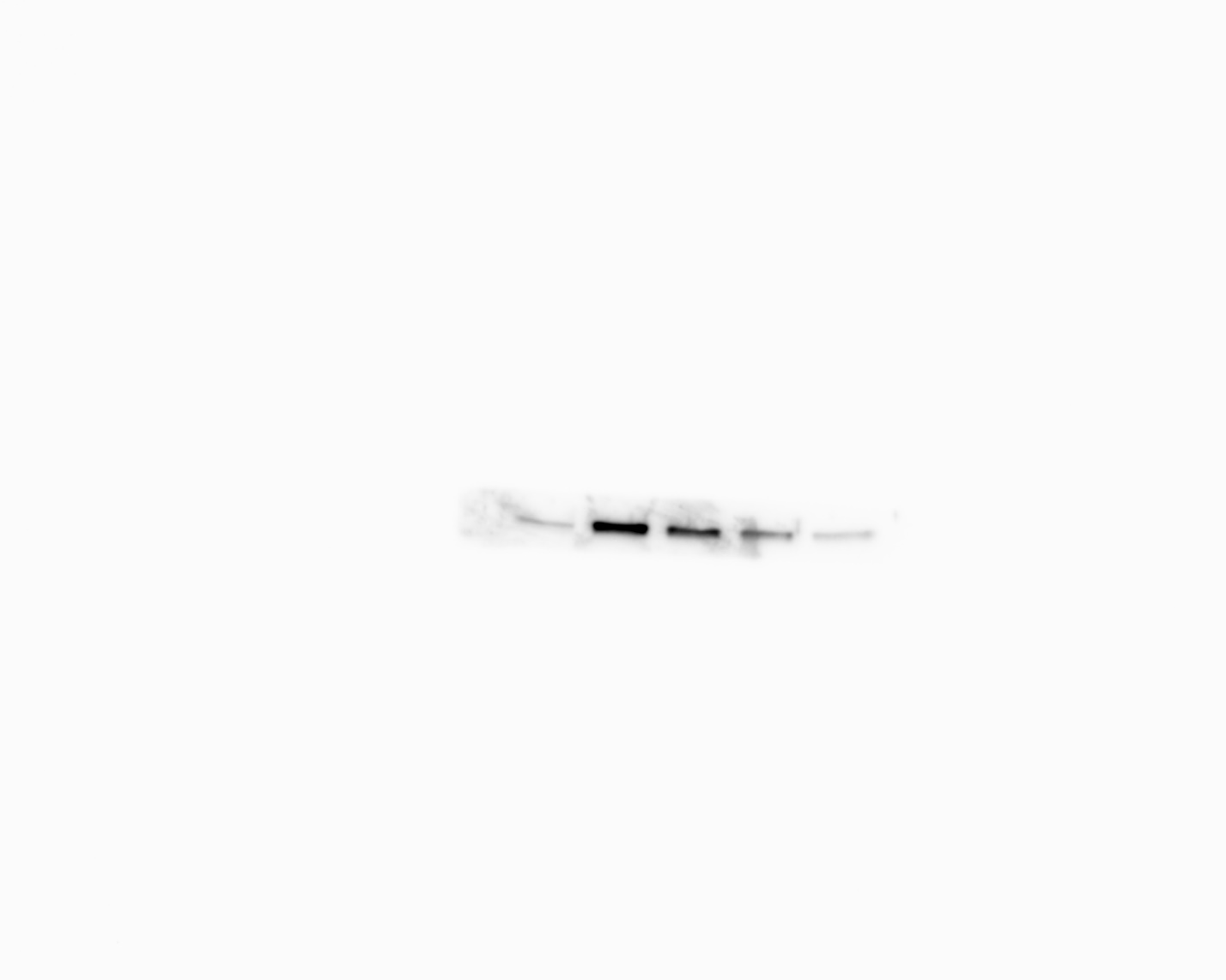


**Fig S8.** Representative western blot image of NP. From left to right: untreated; 12.5 µg/mL; 25.0 µg/mL; 50.0 µg/mL; positive control.


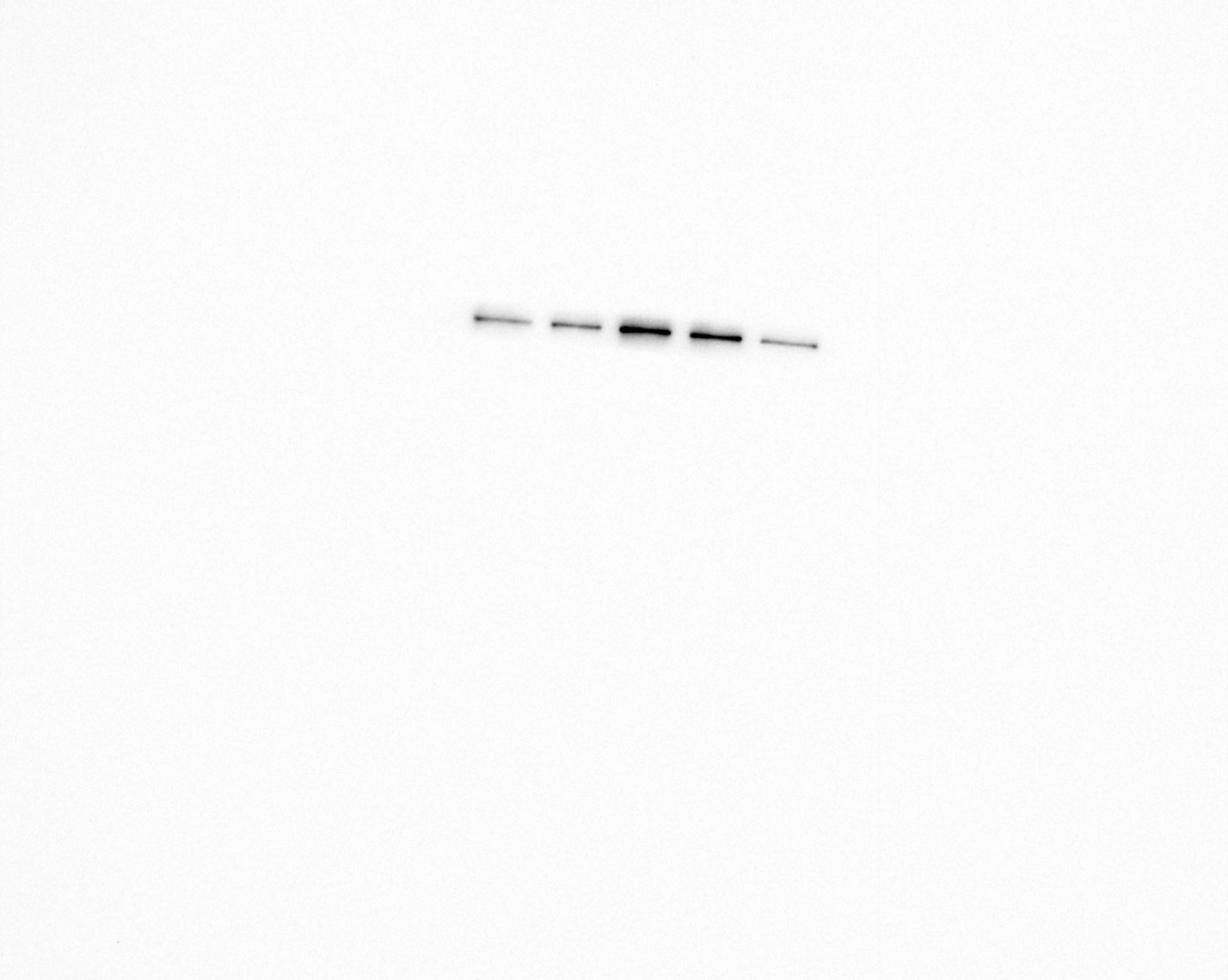


**Fig S9.** Representative western blot image of vinculin. From left to right: untreated; 12.5 µg/mL; 25.0 µg/mL; 50.0 µg/mL; positive control.


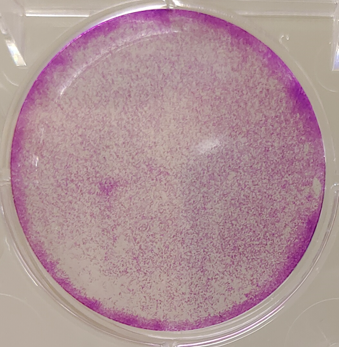

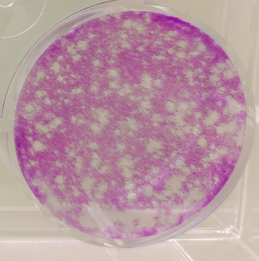

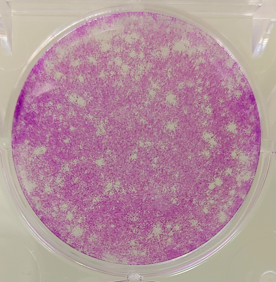

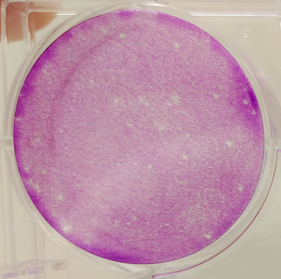

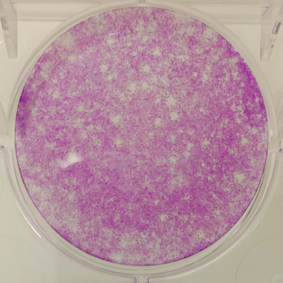

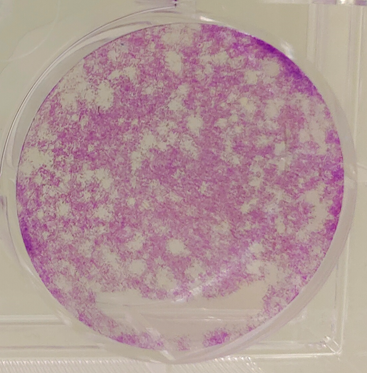

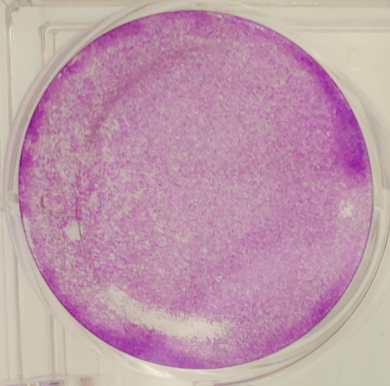


**A**

**B**

**C**

**D**

**E**

**F**

**G**

**Fig S10. Representative images of plaques formed from MDCK cells after supernatant infection. (A) – (C) are from the 48 h treatment group. (D) – (F) are from the 72 h treatment group.** Supernatant from the untreated group **(A)**. Supernatant from the 50.0 µg/mL betacyanin fraction **(B)**. Supernatant from the positive control group **(C)**. Supernatant from the untreated group **(D)**. Supernatant from the 50.0 µg/mL betacyanin fraction **(E)**. Supernatant from the positive control group **(F)**. Mock-infected **(G).** The 48 h group was infected with supernatant diluted at 10^-1^, while the 72 h group was infected with supernatant diluted at 10^-0.301^. A549 cells were first seeded in a 12-well plate and then infected at an MOI of 1 with IAV. The cells received various concentrations of betacyanin after infection for 24 h. The mock-infected group received virus growth medium only without virus. The positive control group received 100 µM of oseltamivir phosphate. The supernatant was harvested after 24 h and was subjected to plaque assay. MDCK cells were seeded in a 6-well plate and then infected with the harvested supernatant at various dilution factors. The cells were then overlaid with an agarose-L-15 medium for 48 h. The agarose overlay was discarded, and cells were stained and fixed with a crystal violet solution. A standard commercial camera was used to photograph plaques formed.


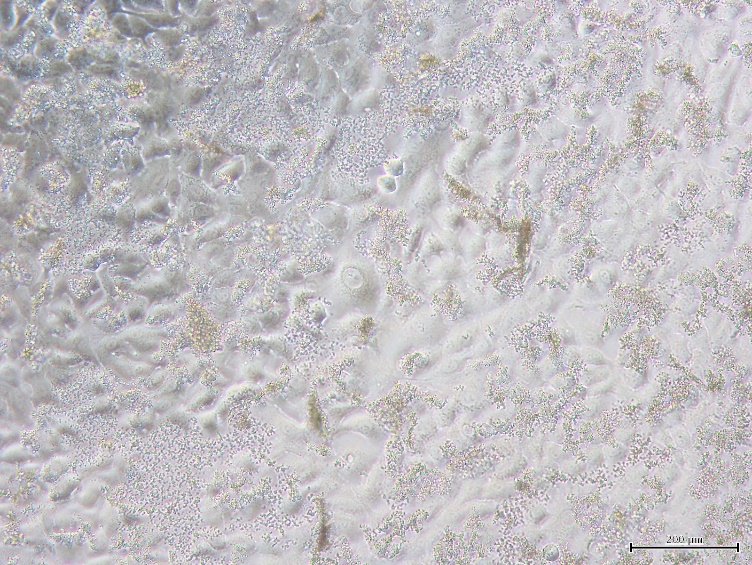

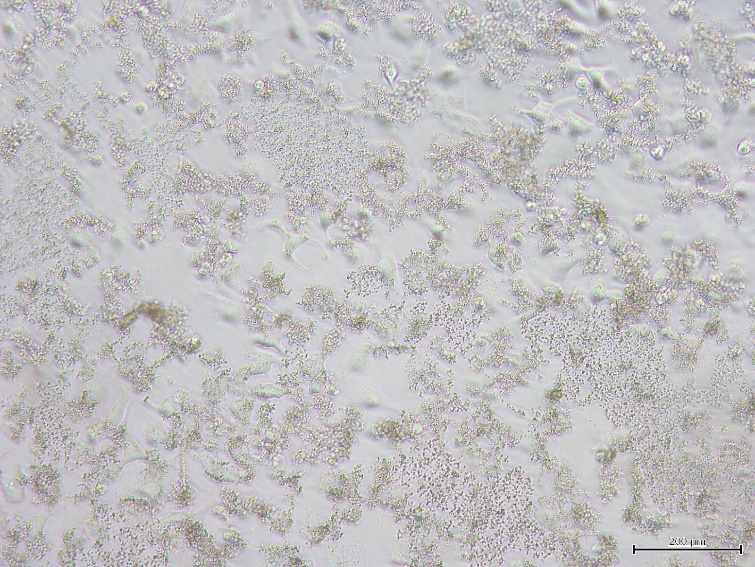

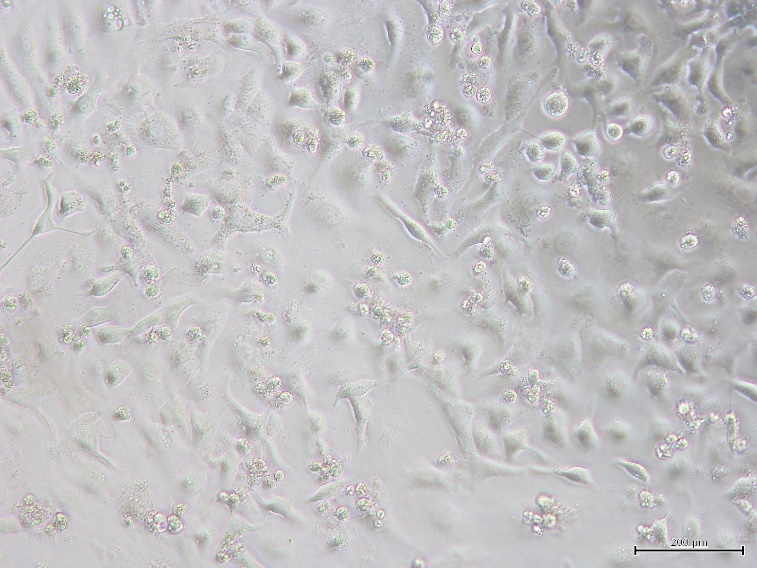

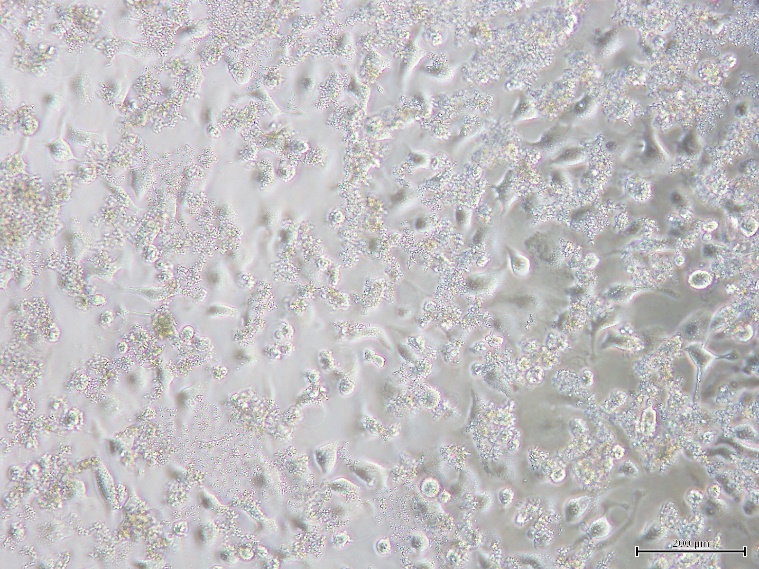


**A**

**B**

**C**

**D**

**Fig S11. Representative images of IAV-infected A549 cells after treatment with 50 µg/mL of betacyanin fraction for 48 hours.** Mock-infected A549 cells **(A)**. A549 cells infected with IAV only **(B)**. IAV-infected A549 cells treated with 50.0 µg/mL betacyanin fraction **(C)**. IAV-infected A549 cells treated with positive control **(D)**. The scale bar represents 200 µm. A549 cells were first seeded in a 96-well plate and then infected at an MOI of 1 with IAV. The cells received 50 µg/mL of betacyanin after infection for 48 h. The positive control group received 100 µM of oseltamivir phosphate. The mock-infected group received virus growth medium only without virus. After 48 h, the cells were visualized with an inverted phase contrast microscope prior to the continuation of the CPE measurement assay.


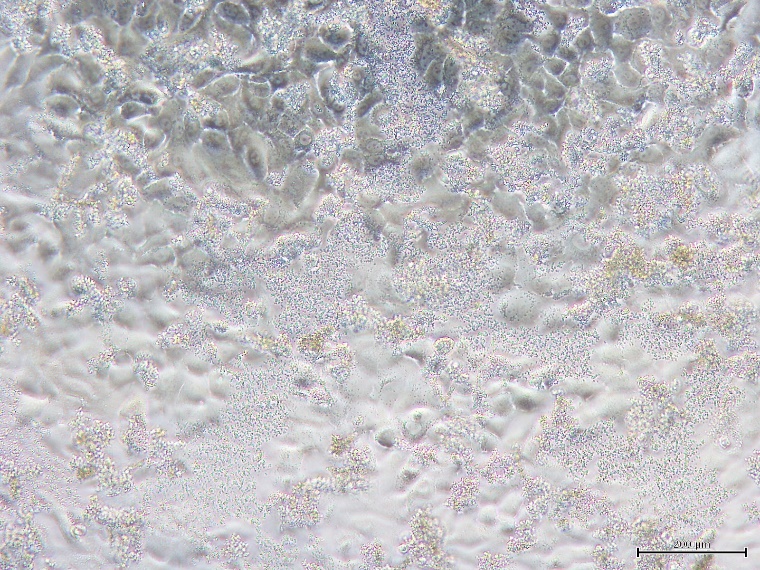

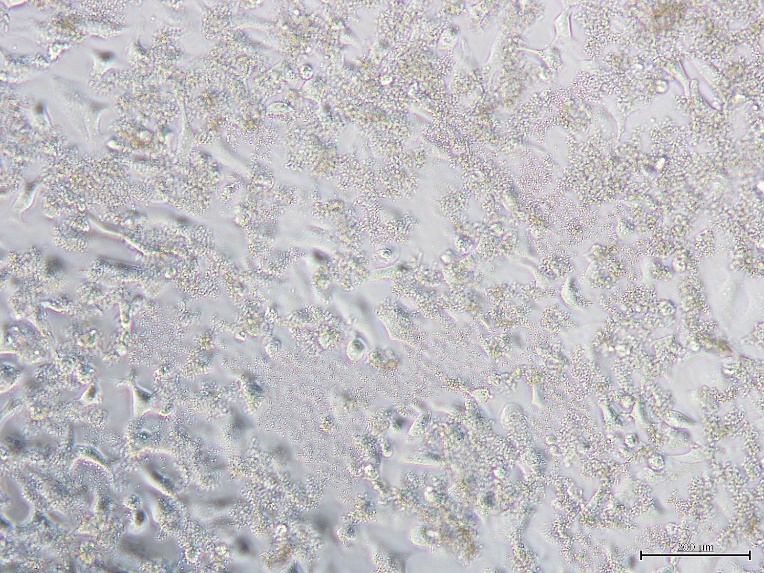

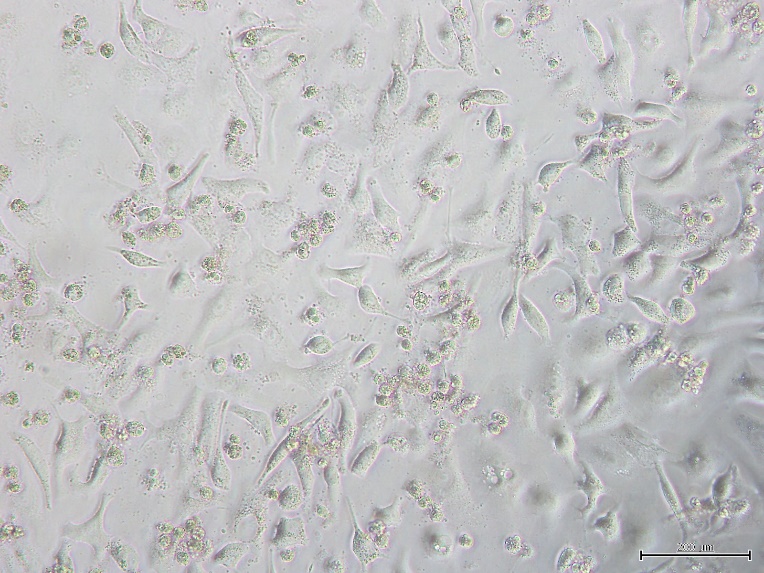

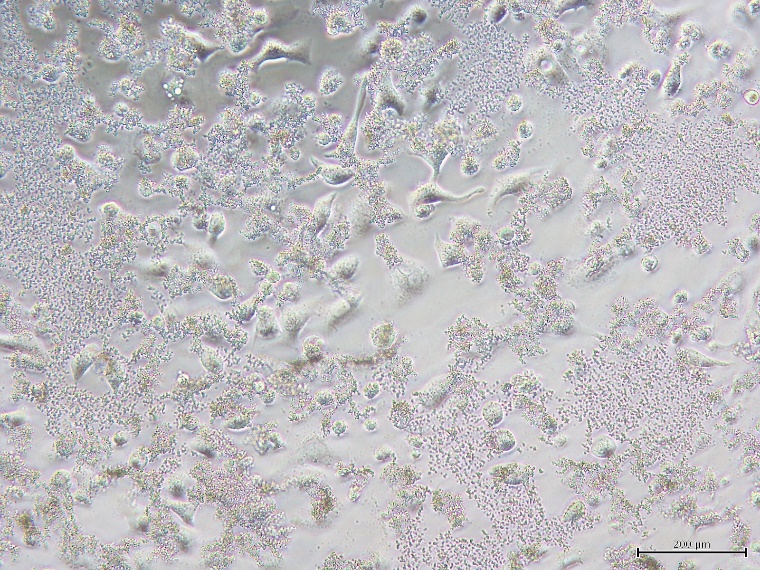


**A**

**B**

**C**

**D**

**Fig** **S12. Representative images of IAV-infected A549 cells after treatment with 50 µg/mL of betacyanin fraction for 72 hours.** Mock-infected A549 cells **(A)**. A549 cells infected with IAV only **(B)**. IAV-infected A549 cells treated with 50.0 µg/mL betacyanin fraction **(C)**. IAV-infected A549 cells treated with positive control **(D)**. The scale bar represents 200 µm. A549 cells were first seeded in a 96-well plate and then infected at an MOI of 1 with IAV. The cells received 50 µg/mL of betacyanin after infection for 72 h. The positive control group received 100 µM of oseltamivir phosphate. The mock-infected group received virus growth medium only without virus. After 48 h, the cells were visualized with an inverted phase contrast microscope prior to the continuation of the CPE measurement assay.
